# Quantitative evaluation of acute myocardial infarction by feature-tracking cardiac magnetic resonance imaging

**DOI:** 10.12669/pjms.39.3.7248

**Published:** 2023

**Authors:** Jun Ye, Wenxia Zong, Xing Wu, Xiaonan Shao, Yue Wu

**Affiliations:** 1 Jun Ye, Department of Radiology, Wuhan No.7 Hospital, Wuhan 430071, Hubei Province, P.R. China; 2 Wenxia Zong, Department of Cardiology, Hubei No.3 People’s Hospital of Jianghan University, Wuhan 430000, Hubei Province, P.R. China; 3 Xing Wu Clinical Laboratory, Xianning Central Hospital, (The First Affiliated Hospital of Hubei University of Science & Technology), Xianning 437100, Hubei Province, P.R. China; 4 Xiaonan Shao, Department of Radiology, Wuhan No.7 Hospital, Wuhan 430071, Hubei Province, P.R. China; 5 Yue Wu, Department of Cardiology, Hubei No.3 People’s Hospital of Jianghan University, Wuhan 430000, Hubei Province, P.R. China

**Keywords:** Feature-tracking cardiac magnetic resonance, Acute myocardial infarction, ST-elevation myocardial infarction, Late gadolinium enhancement

## Abstract

**Objective:**

To assess the value of feature-tracking cardiac magnetic resonance (FT-CMR) imaging in the quantitative evaluation of acute myocardial infarction (AMI).

**Methods:**

We retrospectively analyzed medical records of patients with acute myocardial infarction (AMI) diagnosed in the Department of Cardiology of Hubei No.3 People’s Hospital of Jianghan University from April 2020 to April 2022, who underwent feature-tracking cardiac magnetic resonance (FT-CMR) examination. Based on the electrocardiogram (ECG) findings, patients were divided into ST-elevation myocardial infarction (STEMI) (*n*=52) and non-STEMI (NSTEMI) (*n*=48) groups. We compared myocardial strain parameters between the two groups and applied the Pearson’s test to reveal any correlations between the left ventricular myocardial strain parameters and the number of late gadolinium enhancement (LGE) positive segments; we assessed the clinical value of FT-CMR for predicting STEMI using a receiver operating characteristic (ROC) curve.

**Results:**

The number of LGE-positive segments in the STEMI group was significantly higher than that in the NSTEMI group. The myocardial radial, circumferential and longitudinal strains in the STEMI group were significantly lower than those in the NSTEMI group (*p*<0.05). The number of LGE-positive segments in patients with AMI negatively correlated with the radial, circumferential and longitudinal strains. The results of the ROC curve analysis showed that radial, circumferential and longitudinal strain values have a diagnostic value for STEMI (*p*<0.05).

**Conclusion:**

FT-CMR, a non-invasive and rapid method for analyzing myocardial strains, has a high diagnostic value for AMI and should be helpful for the prevention and intervention of ventricular remodeling after myocardial infarctions.

## INTRODUCTION

The main pathological changes in acute myocardial infarction (AMI) are acute myocardial ischemia and necrosis.^1^ AMIs can be divided into ST-elevation myocardial infarction (STEMI) and non-STEMI (NSTEMI) according to ECG findings.^2^ Both syndromes are caused by severe coronary artery stenosis or complete occlusion (mainly due to thrombosis on atherosclerotic plaques), and have similar clinical manifestations.^3^ Although clinically similar, these conditions differ in the severity of complications, treatment and prognosis.^4^ Therefore, accurate diagnosis of STEMI and NSTEMI is crucial.

Cardiac magnetic resonance (CMR), a non-invasive and radiation-free imaging technology, has become the gold standard for the measurement of cardiac parameters, and feature-tracking cardiac magnetic resonance (FT-CMR) is often used to measure cardiac structures and function (coronary artery imaging, ventricular wall motion, hemodynamics, myocardial activity, *etc*.).^5^-^7^ However, predicting myocardial damage and three-dimensional functional changes of cardiac stress in STEMI patients in advance is impossible.^8^ FT-CMR imaging has been frequently used to detect cardiac stress.^9^ Relevant studies have confirmed that this technology based on a steady-state free precession (SSFP) film sequence of magnetic resonances has a high clinical diagnostic value in cases of severe aortic stenosis, cardiac amyloidosis, heart failure, arrhythmogenic right ventricular dysplasia, cardiomyopathy and other heart diseases.^10^,^11^ However, the reports about the predictive ability of FT-CMR in STEMI are scarce. Therefore, we reviewed the FT-CMR imaging data of patients with AMI, analyzing the correlation between FT-CMR myocardial strain parameters and STEMI, and assessed the predictive ability of FT-CMR for STEMI.

## METHODS

We selected the records of one hundred patients (54 men and 46 women) with AMI diagnosed in the Department of Cardiology of Hubei No.3 People’s Hospital of Jianghan University from April 2020 to April 2022 who underwent FT-CMR examinations. Patients were continuously monitored by electrocardiogram to observe changes in heart rate, heart rhythm, blood pressure, and respiration, and venous access was established soon after admission. Drug and reperfusion therapy were performed according to the patients’ conditions. The disease course ranged from three to six years with an average of 4.46 ± 0.68 years. All the patients received coronary angiography and percutaneous coronary intervention, followed by FT-CMR. The median door-to-ECG, artery access and balloon times were 19 minutes, 84 minutes and 118 minutes, respectively. The average age of the patients was 65.88 ± 8.56 years. The mean left ventricular ejection fraction (LVEF) was 42.56 ± 6.98%. According to ECG records, we had 52 patients with STEMI in the STEMI group, and 48 patients without STEMI in the NSTEMI group. We counted 365 LGE-positive segments of which 251 were detected in the STEMI group and 114 in the NSTEMI group.

### Inclusion criteria:

- Age≥18 years
- Patients with AMI diagnosed by coronary angiography or CT angiography according to the relevant diagnostic criteria formulated by the Chinese Society of Cardiology of Chinese Medical Association:^12^ 1) Elevated serum levels of cardiac isoenzymes or troponin; 2) Persistent chest pain that lasts ≥ 30 minutes and unresponsive to nitroglycerin; 3) ST-segment elevation on electrocardiogram (ECG)
- Patients received FT-CMR examination within one month after AMI onset
- Patients with complete and continuous cardiac imaging data

### Exclusion criteria:

- Presence of other heart diseases, such as heart valve disease, congenital heart disease, cardiomyopathy, or severe arrhythmia
- Severe metabolic diseases of the lungs, liver or kidneys
- Presence of autoimmune diseases
- Patients complicated with cardiac rupture, ventricular wall tumor, appendage thrombosis, heart failure and cardiogenic shock
- Patients who have experienced cardiac arrest

### Ethical Approval:

This study was approved by the medical ethics committee of Hubei No.3 People’s Hospital of Jianghan University (approval number, 2021003; 24^th^ of May, 2021)

### Inspection method:

*Scanner:* We used a 3Tesla MRI Scanner (Discovery MR750; GE Healthcare, Milwaukee, USA) with a steady-state free precession (SSFP) cine sequence, 8-Channel cardiac phased coil, and MR compatible ECG gating and respiratory gating board. *Scanning methods:* 1) We performed axial, coronal and sagittal localization scans with the patient in a supine position. 2) The long axial plane of the left ventricular two-chamber heart and four chamber heart, and the short axial plane of left ventricle were determined on the basis of the standard axial plane. 3) The images were collected to obtain the SSFP film sequence of each section at the end of an expiration.

***We set the following acquisition parameters^13^-^15^:*** setting - turning angle (47.5° ± 2.5), visual field (36.0 cm × 38.0 cm), echo time (1.6 ± 0.1 ms), repetition time (3.2 ± 0.25 ms), and the specified scanning acquisition layer thickness (8 mm). We applied the sensitivity reversion method to measure the two-chamber center, the four-chamber center and the short-axis cavity after 10 to 15 minutes. The imaging parameters considered included the visual field (36.0 cm × 38.0 cm), the repetition time (4.1 -4.2 ms), the echo time fixed (1.6 ms), the specified layer thickness (0.80 cm), the total imaging scanning (8-10 layers), and the set reversal time (300 ms). 4) For the delayed enhancement scan we applied an intravenous injection of gadopentetate dimeglumine (Bayer, Germany; specification: 469.01 mg/ml × 15 ml; approval number: J20080046), the dose was 0.2 mmol/kg, the injection rate 4.0 mL/s, and the same amount of 0.9% sodium chloride was injected at the same rate. After the intravenous injection of contrast agent for 10 minutes, the scanner set with sensitivity inversion recovery sequence took LGE images of short axis, two chamber heart, four chamber heart and left ventricular inflow and outflow tract.

### Image processing scheme:^13^

**Fig.1:**
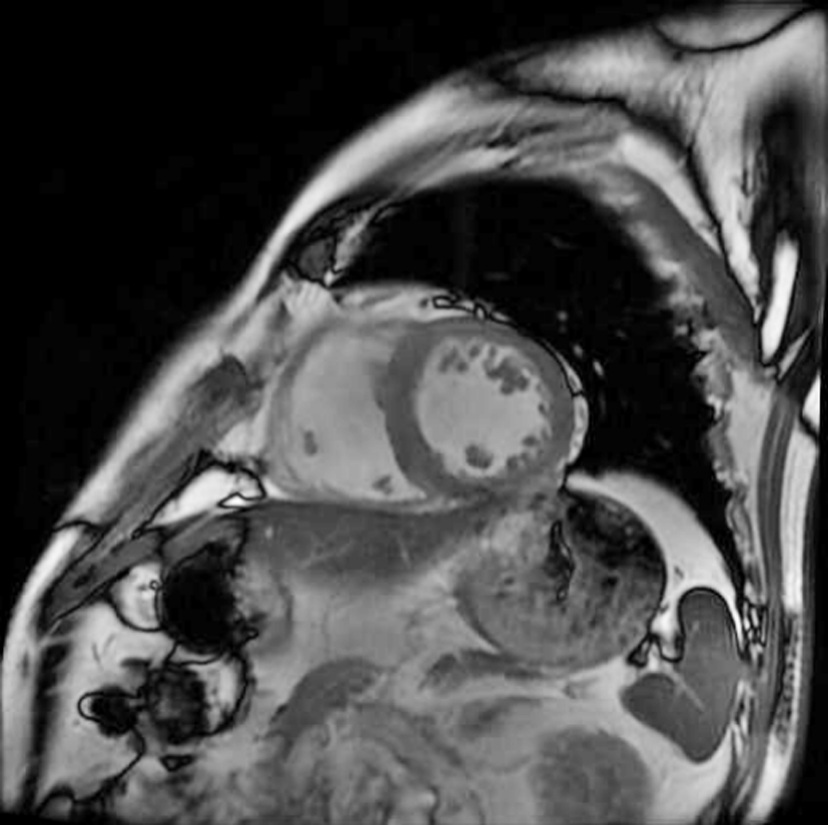
Left ventricular short axis position.

**Fig.2:**
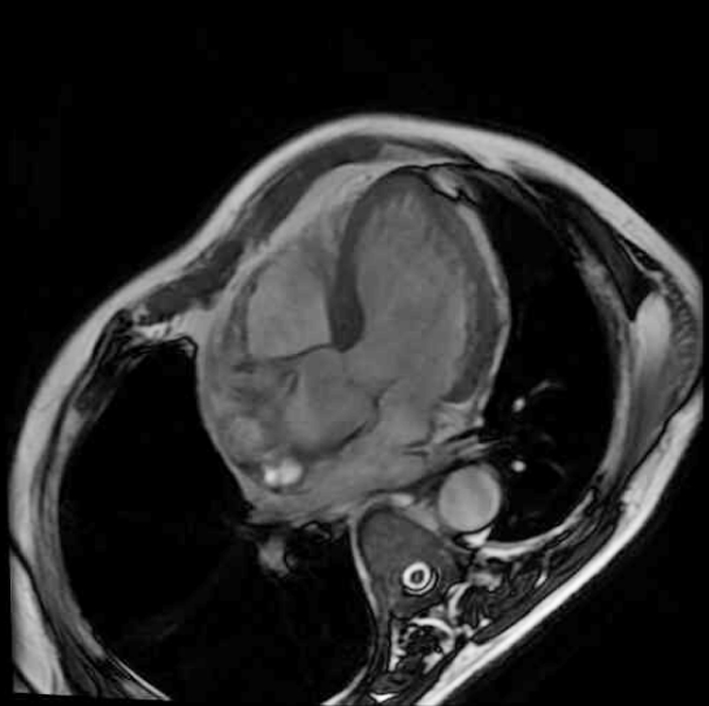
Left ventricular long axis position.

A senior radiologist with a CMR certification blinded to the patient’s condition performed the imaging examination and interpretation of the results. According to the SSFP sequence, the FT-CMR machine generates short axis movies of the two-chamber heart and four-chamber heart and transmits all images to the CVI42 software (circle cardiovascular imaging, Calgary, Canada); the complete cardiac cycle is thus outlined. The left ventricular ejection fraction (LVEF) and myocardial strain parameters (including radial strain, circumferential strain, longitudinal strain, and others) were obtained using the relative movement and displacement of the left ventricular endocardium and the upper edge of the epicardium. The radial strain, circumferential strain, longitudinal strain, and other indexes are compared in absolute values (positive values indicate myocardial lengthening and thickening, negative values indicate myocardial shortening and thinning). Global left ventricular deformation force: for the strain analysis of left ventricular short axis (Fig.1 and long axis view (Fig.2). We divided the left ventricle into 16 segments for evaluation following the American Heart Association standard.^16^ We defined the LGE-positive segment (myocardial infarction segment) as one with a signal intensity larger than five standard deviations from the distal normal myocardium at the same level.

### Statistical Analysis:

We used the SPSS 22.0 software to analyze the data. The measurement data were expressed as mean and standard deviations (*χ̅*±*S*), we applied independent sample *t*-tests for comparisons between groups; count data were expressed as numbers (percentages %), and Chi squared tests were used for comparisons between groups. Pearson’s correlation analyses were used to analyze correlations between the strain values of the left ventricular myocardium and the number of LGE-positive segments. The clinical value of myocardial strain parameters in the diagnosis of STEMI was assessed by analyzing the working characteristics of subjects (in ROC curve).

## RESULTS

A total of one hundred patients diagnosed with AMI were included in this study. In the STEMI group, there were 52 patients (mean [SD] age, 65.13 [8.03] years), 32 (61.54%) males and 20 (38.46%) females; in the NSTEMI group, there were 48 patients (mean [SD] age, 65.87 [8.32] years), 26 (54.17%) males and 22 (45.83%) females. We found similar numbers of cases, ages, sex ratios, disease courses and LVEFs between the two groups (*p*>0.05). However, the number of LGE-positive segments in the STEMI group was significantly higher than that in NSTEMI group (*p*<0.05) (Table-I). The radial, circumferential and longitudinal strains of the STEMI group were significantly lower than those of the NSTEMI group (*p*<0.05) (Table-II).

**Table-I:** Comparison of clinical baseline data between the two groups.

Group	n	Male/Female	Age (years)	Course of disease (years)	LVEF/%	LGE positive segments
STEMI-Group	52	32/20	65.13±8.03	4.53±0.70	41.35±6.39	4.83±1.08
NSTEMI-Group	48	26/22	65.87±8.32	4.62±0.79	42.13±6.79	2.37±0.89
*χ*^2^/*t*		0.557	0.452	0.582	0.596	12.332
p-Value		0.456	0.652	0.562	0.553	<0.001

**Table-II:** Comparison of left ventricular myocardial strain parameters between the two groups.

Group	n	Radial strain (%)	Circumferential strain (%)	Longitudinal strain (%)
STEMI-Group	52	17.07±6.39	-8.70±2.62	-7.58±1.82
NSTEMI-Group	48	21.25±6.61	-12.52±3.33	-11.36±4.07
T		3.217	6.328	6.063
p-Value		0.002	<0.001	<0.001

**Fig.3:**
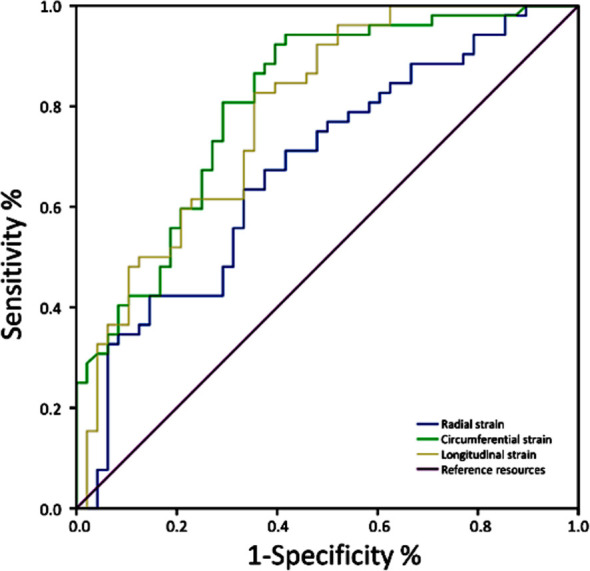
ROC curve of CMR-FT in predicting clinical value of STEMI.

The number of LGE-positive segments in patients with AMI negatively correlated with the radial, circumferential, and longitudinal strains (*p*<0.05) (Table-III) ROC curve analysis showed that the radial strain, circumferential strain and longitudinal strain of left ventricular myocardium had diagnostic value for STEMI (*p*<0.05) (Table-IV) and Fig.3.

**Table-III:** Pearson test for correlation between myocardial strain parameters and LGE in AMI.

Strain	r-Value	p-Value
Radial strain (%)	-0.263	0.008
Circumferential strain (%)	-0.313	0.002
Longitudinal strain (%)	-0.450	<0.001

**Table-IV:** ROC curve parameters of CMR-FT for predicting clinical value of STEMI.

Strain	AUC	Sensitivity	Specificity	95% CI	p-Value	Cut-off Value
Radial strain (%)	0.677	98.10%	12.70%	0.572~0.783	<0.001	28.89%
Circumferential strain (%)	0.866	94.20%	52.50%	0.797~0.935	<0.001	-13.27%
Longitudinal strain (%)	0.788	96.20%	44.10%	0.699~0.877	<0.001	-10.79%

## DISCUSSION

This study analyzed the diagnostic efficacy of FT-CMR and related post-processing techniques for STEMI in patients with AMI. Our results show that the number of LGE-positive segments of the STEMI group was significantly higher than that of the NSTEMI group, indicating that the infarcted area in patients with STEMI was significantly higher than that of patients with NSTEMI, which is consistent with the findings of Dong et al.^17^ FT-CMR can determine whether a stenosis focus is present in different levels of blood vessels, and it can help locate the lesion, its scope, severity and vascular wall stenosis condition, providing a basis for the formulation of an operation plan.^18^ Dannenberg V et al.^19^ conducted an FT-CMR evaluation on 70 patients with STEMI, their results showed that the left ventricular strain parameters are significantly correlated with the risk area and infarct area measured by CMR. In addition, Schuster A et al.^20^ in a meta-analysis study involving 1235 patients with AMI showed that the left atrium peak reservoir strain obtained by FT-CMR has a high prognostic value for predicting major adverse cardiovascular events.

Our results show that the radial, circumferential and longitudinal strains of the left ventricular myocardium in patients with STEMI were significantly lower than those in patients with NSTEMI (p<0.001). This suggests that the epicardial myocardial ischemia injury in patients with STEMI is more serious than that in patients with NSTEMI, a finding consistent with the pathological characteristics of STEMI myocardial infarction involving the whole myocardium.^21^ FT-CMR technology reveals the myocardial strain parameters through post-processing with the strain as a vector, its positive value indicates myocardial lengthening and thickening, and its negative value indicates myocardial shortening and thinning; myocardial strain parameters can quantitatively evaluate myocardial length changes.^22^ Neisius U et al.^23^ used FT-CMR to detect myocardial deformations in 244 consecutive patients and were able to distinguish between hypertensive heart disease and hypertrophic cardiomyopathy. Lai W et al.^24^ studied early CMR myocardial tissue characteristics in 420 patients with first-time STEMI and in 40 patients without obvious heart disease (the control group). Their results showed that the strain values of left and right ventricles in patients with STEMI were significantly lower than those in controls. Our results are consistent with these studies, although we did not observe myocardial strain changes in the right ventricle of the patients with STEMI (a shortcoming of this study). A consistency test for STEMI and LGE-positive segments showed a correlation in patients with AMI. Podlesnikar T et al.^25^ used FT-CMR to evaluate 191 patients one week and six months after STEMI, and showed that the left ventricular infarct area was closely associated with the myocardial strain. Further, ROC curve evaluation showed that the circumferential and longitudinal strains have a high diagnostic value for STEMI (the radial strain has a general diagnostic value). The clinical research results of Bratis K et al.^26^ also showed that in asymptomatic patients with systemic sclerosis and normal routine functional indexes, FT-CMR can identify subclinical insertion fibrosis and/or myocardial infarction based on the left ventricular longitudinal strain damage. These results suggest that during the early stages of myocardial infarction, an FT-CMR film sequence examination without contrast agent, combined with myocardial strain measurements, can help clinicians to quickly identify chest pain causes, predict whether patients with AMI have STEMI, reveal the infarct scope and the arteries involved, provide imaging support to prevent adverse cardiovascular events, avoid reception delays, and reduce the incidence of adverse clinical prognoses.^24^-^26^

### Limitations:

Our study was mainly a retrospective case-control study with a small sample size, without in-depth studies on the impact of FT-CMR on LGE negative sites or the number and location of culprit vessels in patients with STEMI. In addition, we did not assess the impact of baseline data such as age or gender, and we did not analyze the peak strain in each direction during systole. The patients in this study had inevitably taken therapeutic drugs, such as anticoagulants, before we collected the images, and that may have altered the study results. Our survey presented data deviations and our conclusions are subjective. Multi-center RCT studies with large sample sizes are needed to explore and confirm our findings.

## CONCLUSION

FT-CMR is a non-invasive and rapid method for analyzing myocardial strain. It has high diagnostic value for AMI and is helpful for the prevention and intervention of ventricular remodeling after myocardial infarctions.

### Authors’ contributions:

**JY** conceived and designed the study.

**WZ, XW, XS and YW** collected the data and performed the statistical analysis.

**JY** prepared the manuscript and is responsible for the integrity of study.

All authors have read and approved the final manuscript.
